# Exogenous Ghrelin Increases Plasma Insulin Level in Diabetic Rats †

**DOI:** 10.3390/biom10040633

**Published:** 2020-04-19

**Authors:** Haba Elabadlah, Rasheed Hameed, Crystal D’Souza, Sahar Mohsin, Ernest A. Adeghate

**Affiliations:** 1 Department of Pharmacology, College of Medicine & Health Sciences, United Arab Emirates University, Al Ain P.O.B. 17666, UAE; h.alabadlah@gmail.com; 2 Cambridge Medical and Rehabilitation Center, Al Ain P.O.B. 222297, UAE; 3 Department of Anatomy, College of Medicine & Health Sciences, United Arab Emirates University, Al Ain P.O.B. 17666, UAE; rasheedshaul2002@gmail.com (R.H.); crystal.dz@uaeu.ac.ae (C.D.); smohsin@uaeu.ac.ae (S.M.)

**Keywords:** exogenous ghrelin, insulin release, diabetes mellitus, immunoelectron microscopy, immunohistochemistry, metabolic parameters

## Abstract

Ghrelin, a 28-amino acid peptide, is a strong growth hormone secretagogue and a regulator of food intake. In addition, ghrelin is thought to play a role in insulin secretion and in glucose homeostasis. A lot of contradictory data have been reported in the literature regarding the co-localization of ghrelin with other hormones in the islet of Langerhans, its role in insulin secretion and attenuation of type 2 diabetes mellitus. In this study, we investigate the effect of chronic ghrelin treatment on glucose, body weight and insulin level in normal and streptozotocin-induced diabetic male Wistar rats. We have also examined the distribution pattern and co-localization of ghrelin with insulin in pancreatic islet cells using immunohistochemistry and immune-electron microscopy and the ability of ghrelin to stimulate insulin release from the CRL11065 beta cell line. Control, non-diabetic groups received intraperitoneal injection of normal saline, while treated groups received intraperitoneal injection of 5 µg/kg body weight of ghrelin (amino acid chain 24–51) on a daily basis for a duration of four weeks. Our results show that the administration of ghrelin increases the number of insulin-secreting beta cells and serum insulin level in both normal and diabetic rats. We also demonstrated that ghrelin co-localizes with insulin in pancreatic islet cells and that the pattern of ghrelin distribution is altered after the onset of diabetes. Moreover, ghrelin at a dose of 10^−6^ M and 10^−12^ M increased insulin release from the CRL11065 beta cell line. In summary, ghrelin co-localizes with insulin in the secretory granules of pancreatic beta cells and enhances insulin production.

## 1. Introduction

Ghrelin, a 28-amino acid peptide, was discovered in 1999 from a few milligram of stomach extract [1,2]. Ghrelin is encoded in pre-pro-ghrelin, which on splicing yields three peptides; a single peptide, a mature peptide and a C-terminal peptide [3]. The third amino acid (Ser3) of ghrelin peptide is modified by n-octanoic acid, which is a unique acyl modification essential for ghrelin’s activity. The word “Ghrelin” is derived from “ghre”, which means “grow” in proto-Indo-European languages, and “relin”, which means release [4] to indicate the role of ghrelin in stimulating growth hormone release [5].

Ghrelin binds to ghrelin receptor or growth hormone secretagogue receptor (GHSR) which belongs to the G-Protein-coupled receptor family [6]. The *GHSR* gene generates GHS-R1a and GHS-R1b isoforms that differ in their carboxyl-terminal. GHS-R1a has seven transmembrane domains, while GHS-R1b lacks the transmembrane domains 6 and 7. Isoform 1a is known to be the active form where ghrelin binds and yields different signal transduction in different cells to exert its effect [7,8,9]. GHS-R is located in both the central and the peripheral nervous systems [10,11,12]. Furthermore, GHS-R was found to be expressed in the thyroid gland, spleen, myocardium and adrenal gland, stomach, small and large intestines, liver, lung, adipose tissue and pancreas, indicating the numerous roles of ghrelin [13,14].

However, ghrelin itself was reported to be expressed predominantly in the fundus of the stomach [15], kidney glomerulus [16], intestine [17], human placenta [18] and in human T cells, B cells and neutrophils [19]. Ghrelin has also been shown to be present in human pancreas [20], where many studies showed that ghrelin co-localizes with insulin in β cells [21], while others revealed the presence of ghrelin in α cells [22]. Ghrelin was also reported in a new pancreatic islet cell, known as the epsilon cell [23].

Since ghrelin has been localized to many body systems, it has since been shown to play a role in the function of many organ systems. It has also been reported that ghrelin is capable of stimulating gastric acid secretion and motility [24]. In addition, ghrelin has been shown to have a potent cardioprotective effect, where it may help in the prevention of heart failure [25,26]. Plasma ghrelin is said to increase dramatically after the onset of advanced renal failure [27], but it is markedly reduced in advanced cancer cases [28].

In fact, it has been shown that ghrelin is implicated in the proliferation and progression of tumors [29]. The involvement of ghrelin in the etiopathogenesis of cancer is further confirmed by the identification of ghrelin variants (In1-ghrelin) in human mammary gland tumors [30]. Moreover, ghrelin and ghrelin receptor were reported to densely populate cancer of the prostate gland [31]. Ghrelin has an important role in many other physiological functions such as learning [32], memory [33], sleeping [34], depression [35], and addiction [36].

In spite of the well-established role of ghrelin in many body systems, its effect on insulin release from the pancreas has been nothing but controversial. Many studies have shown that ghrelin inhibits glucose-stimulated insulin release from both human as well as rodent models of diabetes mellitus [37,38]. In contrast, Tong and others [39] reported that unacylated ghrelin did not alter basal or glucose-induced insulin release in humans. To further increase the controversy, studies reported by Granata et al. [40] showed that both acylated ghrelin and unacylated ghrelin can increase the level of insulin in experimental diabetes.

These differences may be due to the type of ghrelin, species, tissues or cell line used. Three types of ghrelin have been used in studies examining the effect of ghrelin on insulin release. Acylated ghrelin stimulates food intake and increases body weight gain, adipose tissue pool and hyperglycemia, via the hypothalamic orexigenic pathway [41]. In contrast, unacylated ghrelin does not stimulate food intake nor induce hyperglycemia [41]. A combination of both molecules has also been used to study insulin release from the pancreas [42].

All of these observations clearly indicate that the role of ghrelin on insulin release is far from definite. Studies from our laboratory, using whole length ghrelin peptide, showed that ghrelin is present in the pancreatic islet of rats and can also stimulate insulin release [42]. The controversy regarding the cellular localization of ghrelin in the pancreas and its role in insulin secretion has been ongoing since the discovery of this important peptide hormone by Kojima et al. in 1999 [1]. The aim of this study is to re-visit and clarify the pattern of distribution of ghrelin in the endocrine pancreas. We also intend to explore whether the chronic administration of ghrelin has any role in the augmentation of pancreatic beta cell number or the prevention of diabetes mellitus, using a rodent model of diabetes where insulin is severely depleted. Co-localization studies were also performed to determine whether ghrelin and insulin are topographically associated.

## 2. Materials and Methods

### 2.1. Animal Models

Male Wistar rats aged eight weeks and weighing 250 g were used in this study. All rats were acquired from the College of Medicine and Health Sciences, United Arab Emirates University. Rats were housed in the Animal House Facility, which was maintained at 22–25 °C, along with a 12-h light/dark cycle. A standard pellet diet with tap water was provided *ad libitum*. All rats were kept in plastic cages with wood shavings throughout the study.

All experiments in this study were carried out based on the guidelines provided by the National Institute of Health for the care and use of experimental animals and approved by the Animal Research Ethics Committee, College of Medicine and Health Sciences, United Arab Emirates University, Al Ain, United Arab Emirates (Animal Ethics Committee approval number A5-14).

### 2.2. Induction of Experimental Diabetes Using Streptozotocin

Streptozotocin (STZ) was used to induce diabetes in rats by a single intraperitoneal injection at a dose of 60 mg/kg body weight [43]. STZ was freshly prepared by dissolving it in citrate buffer (0.5 M, pH 4.5). Rats were considered diabetic if their fasting blood glucose level was above 126 mg/dl.

### 2.3. Experimental Design

Experimental rats were distributed randomly into 5 groups:

- Normal control (n = 6): Normal male rats were treated with 5 µg/kg body weight of saline.
- Normal treated (n = 6): Normal male rats were treated with 5 µg/kg body weight of ghrelin (amino acid chain 24–51).
- Diabetic control (n = 6): Diabetic male rats were treated with 5 µg/kg body weight of saline.
- Diabetic Treated (n = 6): Diabetic male rats were treated with 5 µg/kg body weight of ghrelin (amino acid chain 24–51).
- Pre-diabetic Treated (n = 6): Normal male rats were treated with 5 µg/kg body weight of ghrelin (amino acid chain 24–51) for 4 weeks before the induction of diabetes on the 5th week to determine whether ghrelin can prevent STZ-induced diabetes.

### 2.4. Fasting Blood Glucose and Body Weight Measurements

Body weight and fasting blood glucose (FBG) were measured at the beginning of the study and followed up once weekly. Animals were fasted for 12 h and blood samples were collected from the tail vein for the measurement of FBG using OneTouch^®^ Ultra^®^ 2 Glucometer (LifeScan, Inc., Milpitas, CA, USA).

#### Glucose Tolerance Test

Before rats were sacrificed, they were fasted for 12 h followed by intraperitoneal injection of 10 mg/kg/BW glucose to measure the glucose tolerance test (GTT). Blood samples were collected from the tail vein of the rats and the blood glucose level was measured at 0 and 30, 60, 120 min, except for the “pre-diabetic treated group”. The experimental animals were weighed at the end of the experiment using a laboratory scale.

### 2.5. Blood and Tissue Collection

Rats were anesthetized using ether. Blood was obtained from the inferior vena cava and kept in two separate tubes (yellow top: gel tube and purple top: contain EDTA). Pancreatic tissue was collected and cut into two parts: one part for immunohistochemistry (IHC) and immunofluorescence (IF) studies and the second for electron microscopy.

### 2.6. Tissue Processing

Pancreatic tissues were embedded in Zamboni’s fixative and McDowell solutions for overnight fixation prior to IHC and IF studies using light microscopy and for electron microscopy, respectively.

#### 2.6.1. Tissue Processing for Immunohistochemistry and Immunofluorescence Study

After overnight fixation in Zamboni’s fixative, pancreatic tissues were dehydrated in a series of graded ethanol concentrations ascending from 70% to 100% ethanol followed by xylene and subsequently paraffin wax at 55 °C. Tissues were embedded in paraffin blocks and sections of 6 µm thickness were sliced using a microtome (Shandon AS325, Kalamazoo, MI, USA), and placed in a water bath for a few seconds at 40 °C. Tissues were placed on gelatin-coated slides, and kept on a hot plate to dry for a minimum of 2 h to enhance the attachment of the sections.

#### 2.6.2. Avidin-Biotin Complex Staining

Pancreatic tissues sections were rinsed in xylene, followed by a rehydration step using ethanol at different concentrations according to a previously published technique [44]. Briefly, slides were washed with phosphate buffer solution (PBS) before retrieval of antigen using citrate buffer (pH 6). The sections were marked using a Dako pen and incubated with 0.3% hydrogen peroxidase-methanol, then protein blocked, before overnight incubation with corresponding primary antibody (insulin anti-guinea pig or ghrelin anti-rabbit) at 4 °C. The next day, the slides were kept at room temperature before being washed with PBS before incubation with the secondary biotinylated antibody (anti-guinea pig IgG for insulin and anti-rabbit IgG for ghrelin) at room temperature. Another wash with PBS preceded streptavidin peroxidase incubation. Sections were washed with PBS followed by 3,3-diaminobenzidine tetrahydrochloride DAB (Sigma-Aldrich Chemie GmbH, Taufkirchen, Germany) in PBS incubation. Slides were washed with distilled water and dehydrated in a series of ascending concentration of ethanol 50%, 70%, 95% and 100% followed by xylene. The slides were finally mounted with DPX (Dibutylphthalate Polystyrene Xylene) neutral mounting medium. An AxioCam HRc digital camera with AxioVision 3.0 software was used to capture images of stained sections (Carl Zeiss, Oberkochen, Germany). Images were then adjusted for contrast and brightness using image J 1.47 V (NIH, Bethesda, MA, USA).

#### 2.6.3. Double Labeling Immunofluorescence Staining

Double labelling immunofluorescence was performed according to a previously established technique [45]. Briefly, slides were immersed in xylene and later in ethanol for rehydration followed by a distilled water wash before citrate buffer treatment. Sections were then washed with PBS and the area of interest was marked with Dako pen, then they were incubated with the protein block followed by an overnight incubation with primary antibody at 4 °C, (Table 1). Slides of tissue sections were washed in PBS before the secondary antibody incubation step (Table 1). Finally, the sections were washed with PBS before mounting with CITI-Fluore media (Science Services GmbH, München, Germany). Sections were examined using AxioCam HRc digital camera with AxioVision 3.0 software (Carl Zeiss, Oberkochen, Germany) fixed with z-plane fluorescence. Images were merged and contrast and brightness were adjusted using image J 1.47 V.

#### 2.6.4. Tissue Processing for Electron Microscopy

Pancreatic tissue samples were washed with 0.1 M phosphate buffer after overnight fixation and processed for electron microscopy according to a previously described method [46]. Specimens were subjected to 1% osmium tetroxide before dehydration in ascending concentrations of ethanol (30%, 50%, 70%, 95%, and 100%), and then immersed in propylene oxide followed by different ratios of propylene oxide and resin (1:1, 1:2 and 1:3) before infiltration with pure resin overnight at 4 °C. Specimens were further embedded in the resin using molds and kept for overnight polymerization in the oven at 60 °C. Blocks of resin were trimmed and 1 µm semi-thin sections were cut using a glass knife on the ultra-microtome. Sections were transferred into a drop of water on the microscope slides using watchmaker’s forceps and toluidine blue was used to stain the tissues, which were later examined with the microscope to locate the islets of Langerhans using light microscopy examination. Resin blocks were further trimmed for ultrathin sectioning using a diamond knife and were placed on copper grids using a wire loop. The grids were placed on filter paper and left to dry.

#### 2.6.5. Post-embedding Immuno-Gold Double Labeling for Transmission Electron Microscopy

Ultrathin sections were processed for double labelled immunoelectron microscopy according to the methods described by Lotfy et al. [47]. Briefly, grids were washed with deionized water before incubation in 10% H_2_O_2_. Grids were then immersed in 0.5 M NH_4_Cl in 0.01 M PBS (pH 7.3) followed by washing buffer (1% BSA and 0.1% Tween-20).

Tissues were blocked using blocking buffer (20% normal goat serum (NGS) diluted in washing buffer) followed by overnight incubation at 4 °C with primary antibody (Ghrelin anti-rabbit 1:100 diluted in PBS, pH 7.3, containing 1% BSA, 0.1% Tween-20 and 5% NGS). Grids were washed with PBS and incubated with blocking buffer before incubation with goat anti-rabbit IgG conjugated to 15 nm gold particles (diluted 1:20 in antibody buffer).

Grids were then rinsed with PBS before overnight incubation with insulin as a second primary antibody (insulin anti-mouse (1:100) diluted in antibody buffer (PBS, pH 7.3, containing 1% BSA, 0.1% Tween-20 and 5% NGS)), which was labeled with 5 nm gold particles diluted in 1:20 antibody buffer before fixation with glutaraldehyde (2.5% aqueous). Grids later were rinsed with deionized water before leaving them to dry on filter paper. Contrasting the grids was done with uranyl acetate and lead citrate. The sections were later viewed with a Philips TEM.

### 2.7. Stimulation of CRL11065 Beta Cell Line with Ghrelin

A total of 2.15 × 10^−6^ cells from CRL11065 beta cell line (American Type Culture Collection, Manassas, VA, USA) were incubated with different concentrations (10^−6^ M–10^−12^ M) of ghrelin according to a previously described method [42]. Briefly, cells were placed in Krebs solution containing 2.8 mM and with either 10^−6^ M, 10^−9^ M, or 10^−12^ M of ghrelin and incubated in a water bath at 37 °C for 1 h. The basal group was incubated with Krebs solution only. The insulin content of supernatant was determined using rat insulin ELISA kit (Cat#: 10-1251-01, Uppsala, Sweden). The number of CRL11065 beta cells used was determined with a cell counter (Countess II, Thermo Fisher, Waltham, MA, USA).

### 2.8. Morphometric Analysis of Ghrelin and Insulin-Positive Cells

The percentage distribution of ghrelin- and insulin-positive cells in pancreatic islets was determined in ghrelin-treated and untreated controls and diabetic rats using Image J software^®^ (NIH, Bethesda, MD, USA). The number of ghrelin- and insulin-positive pancreatic islet cells was counted and divided by the total number of islet cells to find their percentage distribution in line with an earlier technique [48]. Five islets were counted for each rat in each group (n = 6).

### 2.9. Morphometric Analysis of Secretory Granules in Pancreatic Beta Cells

The total number of secretory granules in pancreatic beta cells of ghrelin-treated and untreated controls and diabetic rats was counted using Image J software^®^. Granules containing both ghrelin and insulin were also determined to assess the degree of co-localization according to a technique previously used in our laboratory [48]. Eight to eleven fields were counted for each rat in each group of six rats.

### 2.10. Statistical Analysis

Statistical analysis was performed using GraphPad Prism version 5.01. Data were presented as mean ± standard deviation (SD). One-way ANOVA was used between experimental groups and corresponding control groups, followed by post-hoc Bonferroni. Unpaired t-test was used to analyze the significance of differences between mean values within the group between two different time points. Values of *p* < 0.05 were considered to be statistically significant.

## 3. Results

### 3.1. Effect of Ghrelin Treatment on Body Weight, Fasting Blood Glucose Level and Glucose Tolerance Test

**Figure 1:**
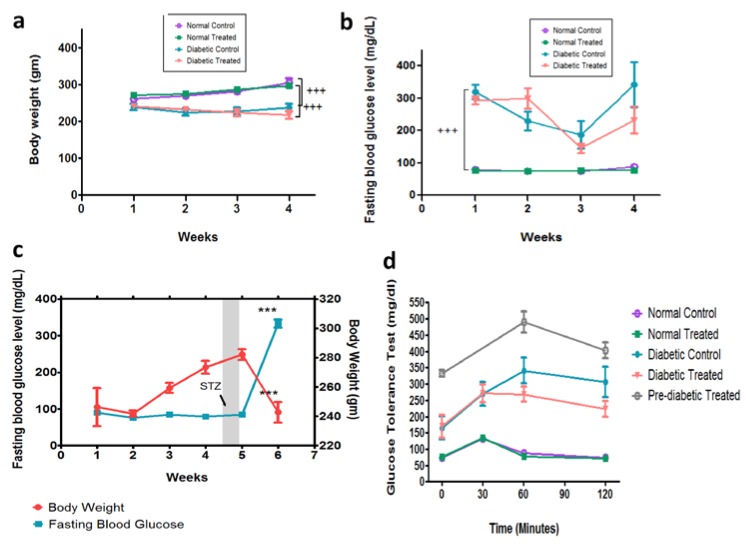
Effect of ghrelin on body weight, fasting blood glucose and glucose tolerance test. (**a**) Effect of ghrelin treatment on body weight (gm) in normal and diabetic Wistar rats treated with either ghrelin or normal saline (NS), during a course of four weeks (n = 7). (**b**) Effect of ghrelin treatment on fasting blood glucose level (mg/dL) in normal and diabetic Wistar rats treated with either ghrelin or normal saline (NS) (n = 7). (**c**) The effect of four weeks of ghrelin treatment on preventing or delaying the onset of STZ-induced diabetes mellitus in Wistar rats (Group V, Pre-diabetic). Fasting blood glucose level “mg/dL” (blue line) and body weight “gm” (red line) in normal rats treated with ghrelin before induction of diabetes in the fifth week of the study (arrow). (n = 7). (**d**) The effect of ghrelin treatment on glucose tolerance test in normal and diabetic groups treated with either ghrelin or NS (n = 7). Results are expressed as mean ± SD. (*** *p* ≤ 0.001).

At the end of the experiment, the body weight of diabetic rats treated with ghrelin did not significantly change compared to that of the corresponding untreated diabetic controls. Moreover, the administration of ghrelin to normal, non-diabetic rats did not significantly alter body weight when compared to that of untreated non-diabetic rats. In contrast, the body weight of treated and untreated normal, non-diabetic rats was significantly higher than that of their diabetic counterparts (Figure 1a). Ghrelin treatment did not significantly reduce FBG level in diabetic rats compared to untreated diabetic controls. The FBG in all diabetic groups (treated and untreated) was significantly (*p* < 0.05) higher compared to those of non-diabetic groups (treated and untreated, Figure 1b). In the pre-diabetic treated group, treating rats with ghrelin prior to the chemical induction of diabetes via STZ resulted in body weight increase, while the FBG was maintained within the normal range (Figure 1b). However, upon diabetes induction using STZ, pre-diabetic treated rats showed a significant reduction in their body weight, whereas fasting blood glucose level was increased significantly (Figure 1c).

#### Effect of Ghrelin Treatment on Glucose Tolerance Test

There was no significant difference in blood glucose levels between ghrelin and normal saline treatment in normal groups. Although not statistically significant, diabetic rats treated with ghrelin exhibited a slight improvement in response to glucose challenge when compared to diabetic group that received normal saline at 60 and 120 min after glucose load (Figure 1d). The pre-diabetic ghrelin-treated group showed elevated blood glucose at baseline when compared to the other two diabetic groups, which can be explained by the new onset of diabetes. The new onset of diabetes after injection of STZ in this group indicates that the administration of ghrelin to normal rats for 4 weeks did not prevent the occurrence of diabetes. However, this group handled glucose challenge well at 120 min after glucose loading (see the slope of decrease). (Figure 1d).

### 3.2. Effect of Ghrelin Treatment on the Pattern of Distribution of Insulin-Positive Cells

**Figure 2:**
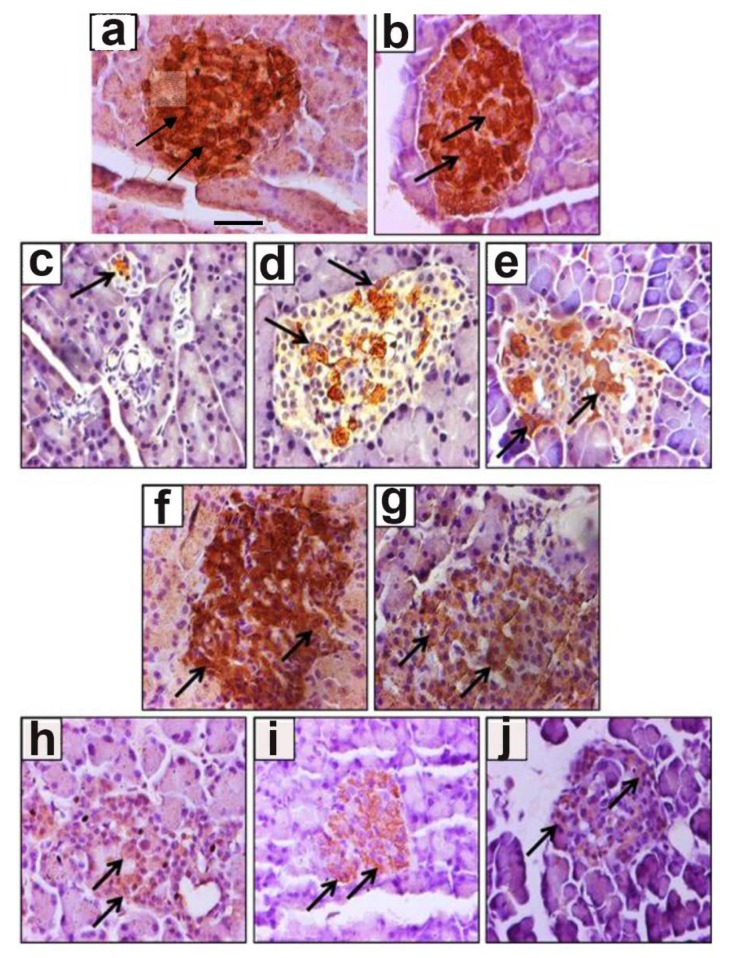
Immunohistochemical localization of insulin and ghrelin in non-diabetic and diabetic rat pancreas. Distribution pattern of insulin-secreting cells (**a**–**e**) and ghrelin-secreting cells (**f**–**j**) in the pancreatic tissue. Normal control (**a**,**f**), normal treated (**b**,**g**), diabetic control (**c**,**h**), diabetic treated (**d**,**i**) and pre-diabetic ghrelin-treated (**e**,**j**). Arrows shows insulin- and ghrelin-immuno-positive cells. Data are typical of those for 4 different animals in each group. Scale bar = 25 µm.

Insulin-positive cells: insulin-immunoreactive islet cells were located mainly in the central and peripheral regions of the islets of normal control rats (Figure 2a). The pattern of distribution of insulin-immunoreactive cells was not altered after treatment of non-diabetic rats with ghrelin (Figure 2b). In the diabetic control group (Figure 2c), insulin-immuno-positive cells were hardly seen in the islet as a result of STZ selective destruction of insulin-secretin β cells. On the other hand, a higher number of insulin-immuno-positive cells can be observed randomly in both the central and peripheral regions of islets of diabetic rats treated with ghrelin before and after the onset of DM, (Figure 2e,d), respectively.

Ghrelin-positive cells: ghrelin-containing cells were observed in the central as well as peripheral regions of pancreatic islets of normal control rats, where insulin-secreting cells are usually found (Figure 2f). Meanwhile, in normal rats treated with ghrelin, the distribution pattern of ghrelin-immuno-positive cells was similar to that of untreated non-diabetic control (Figure 2g). It was observed that not only is the number of insulin-secreting cells affected by DM (Figure 2c), but also the number of ghrelin-containing cells appears to be reduced when compared to that of normal control (Figure 2h).

Diabetic rats treated with ghrelin after the onset of diabetes showed an intact distribution pattern of ghrelin-containing cells similar to those in normal control (Figure 2i), while ghrelin-immuno-positive cells are few and randomly distributed in pancreatic islets of pre-diabetic ghrelin-treated rats, which is similar to what was observed in the diabetic control group (Figure 2j).

**Figure 3:**
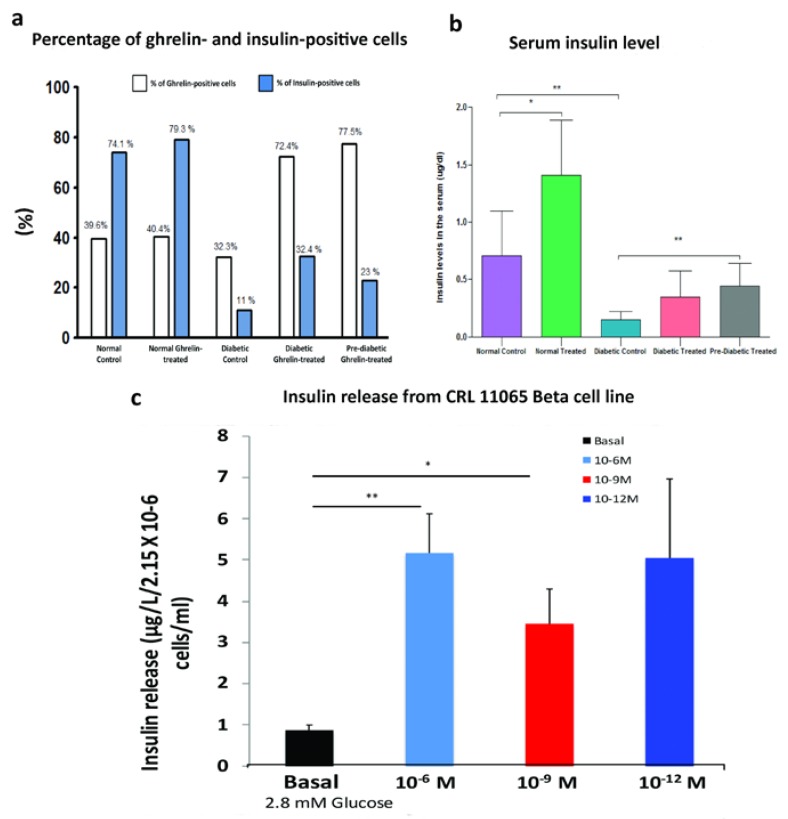
Distribution of ghrelin and insulin in pancreatic islets and insulin level in the serum and from pancreatic beta cell line after ghrelin treatment; (**a**) percentage of insulin- and ghrelin-immuno-positive cells; (**b**) quantitative determination of insulin level in the serum of normal and diabetic groups treated with either ghrelin (before and after diabetes onset) or normal saline (NS), for a course of four weeks (n = 4–7). Results are expressed as mean ± SD. * *p* < 0.04 and ** *p* < 0.02; (**c**) effect of ghrelin on insulin release from CRL11065 beta cell line. (n = 4–7). Results are expressed as mean ± SD * *p* < 0.03 and ** *p* < 0.01. Unpaired *t*-test.

Morphometric analysis showed that ghrelin treatment increased the number of insulin-producing cells in pancreatic islets of normal, untreated rats compared to normal control rats. Meanwhile, in diabetic control (untreated), the percentage distribution of insulin-containing cells showed dramatic reduction compared to the normal control. In contrast, ghrelin treatment in diabetic and pre-diabetic groups resulted in a significant (*p* < 0.05) increase in insulin-positive cells when compared to diabetic control (Figure 3a). In contrast, ghrelin treatment did not significantly increase the percentage distribution of ghrelin-immuno-positive cells, in either normal treated or diabetic treated rats compared to their corresponding controls (Figure 3a).

### 3.3. Effect of Ghrelin on Serum Insulin Level

STZ-induced DM was associated with a significant (*p* < 0.02) reduction in serum insulin level when compared to normal control rats. However, ghrelin treatment resulted in a marked (*p* < 0.04) increase in insulin serum levels in normal treated and diabetic treated (before and after the onset of diabetes) groups in comparison to their corresponding controls (Figure 3b).

### 3.4. Effect of Ghrelin on Insulin Release from CRL11065 Beta Cells

Stimulation of CRL11065 beta cells with either 10^−6^ or 10^−9^ M of ghrelin induced large and significant (*p* < 0.01–0.04) increases in insulin release. Although the quantity of insulin released from CRL11065 beta cells after stimulation with 10^−12^ M of ghrelin was much larger than that of the basal, there was no significant difference because of the high standard deviation (Figure 3c).

### 3.5. Co-Localization of Ghrelin with Insulin in Pancreatic Islet Cells

#### Ghrelin and Insulin

**Figure 4:**
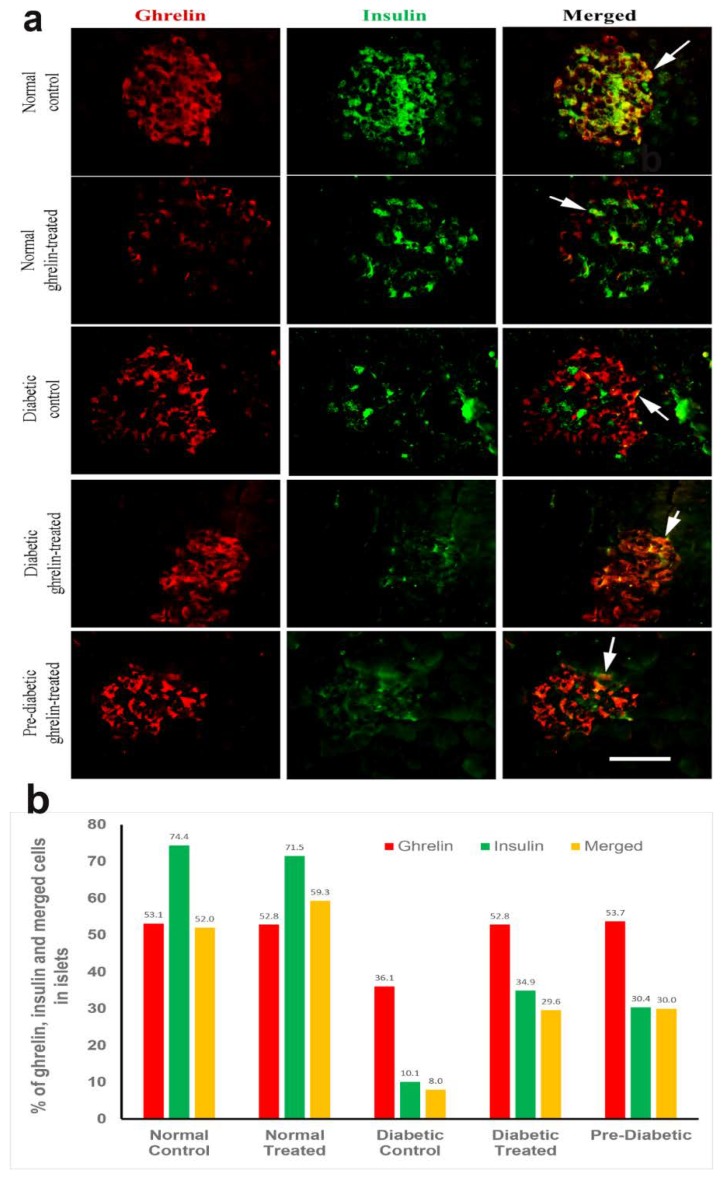
Co-localization of ghrelin with insulin in pancreatic islet cells. (**a**) Ghrelin-containing cells (red), insulin-containing cells (green) and the merged image (red-green-yellow) where co-localization of ghrelin and insulin (white arrows) can be seen. n = 6. Scale bar = 25 µm; (**b**) Percentage distribution of ghrelin- and insulin-immunoreatcive cells in normal control, normal treated, diabetic control, diabetic treated rats and normal rats treated with ghrelin for 4 weeks before the induction of diabetes (pre-diabetic group). Note that the percentage distribution of ghrelin increases with the onset of diabetes mellitus compared to that of insulin.

Double-labelled immunohistochemistry was performed to determine whether ghrelin, observed in both the peripheral and central parts of pancreatic islet, co-localizes with insulin. Our results show that in ghrelin-treated and untreated non-diabetic control rats, ghrelin localizes in both the central and peripheral portions of the islet, where insulin-secreting cells are usually found. The merging of the images stained for ghrelin with those stained for insulin shows that ghrelin is co-localized with insulin in pancreatic islets (Figure 4).

In diabetic groups, ghrelin-positive cells appear numerous in the pancreatic islet, in contrast to insulin-positive cells, which are observed to be fewer. Moreover, the distribution pattern of insulin secreting cells is disrupted due to DM. On the other hand, ghrelin-positive cells localize mainly in the central portion of the pancreatic islet. On merging, cells showed co-localization between ghrelin and insulin (white arrows) (Figure 4a,b).

### 3.6. Immuno-Electron Microscopy of Ghrelin in Pancreatic Beta Cells

**Figure 5:**
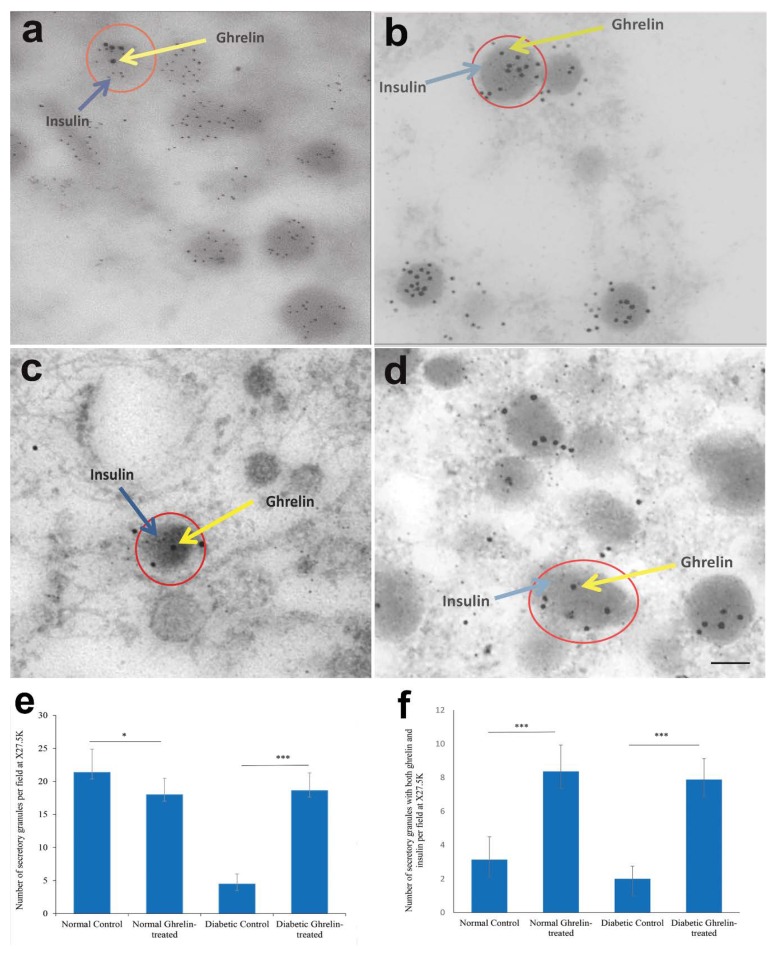
Electron micrographs showing immunogold labeling of ghrelin (15 nm gold particles) and insulin (5 nm gold particles) in the secretory granules of pancreatic beta cells of Wistar rats. (**a**) non-diabetic normal control; (**b**) normal non-diabetic rats treated with ghrelin; (**c**) untreated diabetic rats; (**d**) diabetic rats treated with ghrelin. Ghrelin-(yellow arrow) and insulin-labelled (blue arrow) nanoparticles in the secretory granules (red circle) of beta cells. Scale bar = 5 µm; n = 6; (**e**,**f**) Morphometric analysis of secretory granules in pancreatic beta cells of non-diabetic and diabetic rats treated with either ghrelin or saline; (**f**) analysis of secretory granules of pancreatic beta cells containing both ghrelin- and insulin-positive particles. n = 8–11. * *p* < 0.01, *** *p* < 0.00007.

Double-labeled immunoelectron microscopy wasperformed to determine the exact ultrastructural localization of ghrelin molecule in pancreatic beta cell. Most of the secretory granules in the pancreatic beta cell of non-diabetic control rats contain insulin, whereas ghrelin is observed with insulin in some granules. Ghrelin and insulin where found to share the same secretory granules in pancreatic beta cells of the pancreas of non-diabetic control rats (Figure 5a). This observation confirms our immunohistochemical result that ghrelin co-localized with insulin in pancreatic beta cells. Ghrelin and insulin were observed to be co-localized in the secretory granules of beta cells of non-diabetic rats treated with ghrelin. A large number of secretory granules of pancreatic beta cells of ghrelin-treated, non-diabetic, rats contain both ghrelin and insulin (Figure 5b).

In contrast, the pancreatic beta cells of untreated diabetic rats contain a fewer number of secretory granules, when compared to that of treated and untreated non-diabetic rats. In spite of this, ghrelin co-localized with insulin in the same secretory granules of the few surviving pancreatic beta cells in the diabetic control (Figure 5c). Ghrelin treatment of diabetic rats resulted in an increased number of secretory granules in pancreatic beta cells. These granules contain both ghrelin as well as insulin (Figure 5d). Morphometric analysis showed that the number of secretory granules in pancreatic beta cells increased significantly in ghrelin-treated rats (Figure 5e) with a concomitant increase in the number of granules containing both ghrelin and insulin (Figure 5f). Moreover, the percentage of secretory granules that contain both insulin and ghrelin-positive nanoparticles depends on the group of rats. This percentage was 5%, 86%, 50%, and 76%, respectively, in normal, normal ghrelin-treated, diabetic control, and diabetic treated rats. All pancreatic beta cells contain granules with insulin-positive nanoparticles; however, not all secretory granules contain both hormones (ghrelin and insulin). We did not find ghrelin in pancreatic alpha or any other cell type.

## 4. Discussion

### Body Weight Gain, Blood Glucose and Glucose Tolerance

The administration of ghrelin to either normal or diabetic rats did not significantly increase body weight gain when compared to their corresponding controls. Although it was initially assumed that ghrelin induces body weight gain by stimulating the urge to take food [49,50], the ability of ghrelin to induce body weight gain is not universal. The availability of genetic models to test different metabolic effects of ghrelin has indeed cast doubt on this assumption [51]. In fact, our result on the inability of ghrelin to induce body weight gain is in accordance with that of Huang et al. [52], who showed that ghrelin did not stimulate body weight gain in wild male mice compared to leptin knockout mice [52]. The differences in the outcome of ghrelin injection on body weight gain would also depend on the species of the animal used, the dosage and the duration of the treatment.

The maintenance of glucose homeostasis is a very complicated but important process. Many neuroendocrine hormones are involved in the regulation of glucose uptake, storage and release. In our study, ghrelin was shown to slightly affect the level of fasting blood glucose in diabetic rats as well as enhancing their ability to handle acute glucose load when compared to their corresponding non-diabetic control group, which implies that ghrelin has an important role in glucose homeostasis. This observation is in agreement with those of Heppner et al. [53]. However, the controversy about the exact effects of ghrelin on blood glucose level remains unresolved [54]. Our results on the effect of ghrelin on blood glucose level corroborate those of Golchoobian et al. [55]. These reports are in contrast to those of Peng et al. [56], who reported that exogenous administration of ghrelin at 10 μmol/kg/day for 2 weeks increased blood glucose in Wistar rats. Again, the difference between our observation and those reported by Peng et al. could be explained by many factors including dose regimens, animal species and many others. Regarding the ability of ghrelin-treated rats to process glucose challenge, we observed that all groups of Wistar rats treated with ghrelin handled glucose challenge better than their corresponding controls.

Although ghrelin cells in the pancreas (epsilon cells) have been reported to represent about 1% of total islet cells [57], our study showed that the percentage of ghrelin-immuno-positive cells is higher in all groups of rats examined. This indicates that ghrelin is also expressed by other types of cells in pancreatic islet in addition to epsilon cells. Moreover, inducing DM via STZ resulted not only in reducing insulin-immuno-positive cells but also ghrelin-immuno-positive cells which led to the assumption that either epsilon cells are also sensitive to STZ or that many of the β cells that were destroyed contained both insulin and ghrelin. Our observation is in accordance with that of Volante et al., who showed that ghrelin co-localizes with pancreatic beta cells in humans [21]. The reported differences in the localization of ghrelin to different endocrine cells of the pancreas may be due to the large variety of models and methods used. In order to further confirm the exact intracellular location of ghrelin in pancreatic islet cells, we performed immunoelectron microscopy of the endocrine pancreas. Our results confirm that ghrelin is indeed localized in the secretory granules of pancreatic beta cells, thereby confirming that ghrelin and insulin are located within the same cytoplasmic organelle and therefore the same cell. This suggests an intimate interrelationship between these two peptide hormones. In addition, the secretory granules of pancreatic beta cells of rats treated with ghrelin contain more ghrelin compared to untreated controls. This indicates that the ghrelin injected intraperitoneally was likely taken up by pancreatic beta cells and incorporated into the secretory granules. However, it is also possible that some or all of the ghrelin may be endogenous because it has been reported that pancreatic cells can also secrete ghrelin [58]. It was intriguing to note that not all secretory granules of pancreatic beta cell contain both insulin- and ghrelin-positive nanoparticles. Although reports have shown that ghrelin is produced by epsilon cells of the pancreas [57,59], we did not find ghrelin-positive granules in other cell types. Other studies have reported that ghrelin co-localized sporadically with glucagon in early and late fetal periods and eventually separate later in adult life [60]. Our study showed a close association with pancreatic beta cells, *albeit* in adult rats.

The results of our study confirm that although large numbers of ghrelin-positive cells co-localize with insulin in pancreatic beta cells, there is a probably a small sub-population of pancreatic islet cells which contain ghrelin only. It also appears from our study that in diabetes, where the number of insulin-positive cells is largely reduced [45], the number of ghrelin-containing cells did not decrease in tandem with that of insulin. Whether these ghrelin-immunoreactive cell are *de novo* cells is yet to be elucidated. In this study, ghrelin treatment and pre-treatment (before the onset of DM) resulted in a significant increase in insulin-containing cells. This finding is in agreement with that of Turk et al., who reported that the number of insulin-positive cells increased after the administration of 100 µg of ghrelin per day for four weeks [61]. It is of interest to note that we were able to elicit an increase in the number of pancreatic beta cells with 5 µg of ghrelin per day for four weeks instead of the 100 µg used by Turk and associates [61].

**Figure 6:**
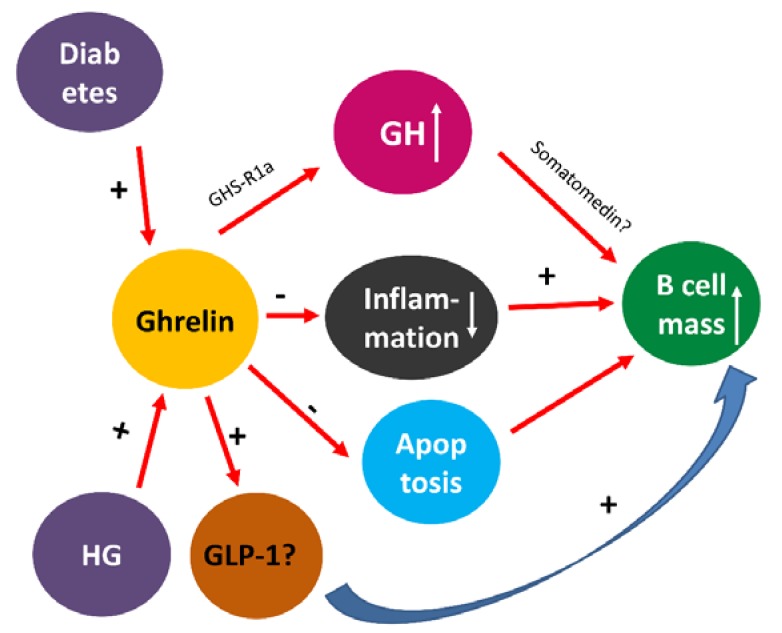
A schematic diagram showing putative pathways by which ghrelin increases pancreatic beta cell mass in diabetes. Diabetes mellitus/hyperglycemia (HG) increases ghrelin release. Ghrelin in turn may either stimulate GLP-1, reduce apoptosis and tissue inflammation or induce the production of somatomedin via increased growth hormone (GH) to enhance beta cell proliferation.

Ghrelin treatment was shown not only to rescue the insulin-secreting cells from STZ selective destruction in diabetic rats but also it appears to enhance either the proliferation or the differentiation of insulin-secreting cells, which resulted in a higher percentage of those cells. The ability of ghrelin to stimulate pancreatic beta cell proliferation has been reported previously. It was reported that ghrelin enhances direct beta cell proliferation in RIP-GG Tg mice [62]. In addition, ghrelin has also been shown to inhibit apoptosis. A recent study on obestatin, a gut peptide that belongs to the ghrelin family, showed that obestatin inhibits apoptosis by reducing the number of TUNEL-positive cells. Since DM is associated with increased hyperglycemia [63,64] which in turn induces the release of inflammatory cytokines [65], the anti-inflammatory effects of ghrelin may help pancreatic beta cells to recover from these noxious events and become more functional. Indeed, ghrelin has been shown to have a strong anti-inflammatory effect in many organs in both experimental and human models of diseases [66,67], which may help pancreatic beta cells to proliferate and be more functional. All of these factors, coupled with its ability to stimulate growth hormone [68], mean that it is therefore logical that ghrelin would be able to stimulate pancreatic beta cell proliferation. Studies have shown that ghrelin can stimulate GLP-1 [69], which in turn increases pancreatic beta cell mass via a large variety of mechanisms [47]. The putative mechanism by which ghrelin stimulates pancreatic beta cell proliferation in depicted in Figure 6.

Furthermore, increasing insulin-secreting cells will logically mean increasing plasma insulin levels, as shown in our study, which also showed that despite the new onset of DM in pre-diabetic treated group, ghrelin pre-treatment induced a significant increase in serum insulin level. The effect of the increase in the serum level of insulin and the number of insulin producing cells in the pre-diabetic group (normal rats received ghrelin before the induction of diabetes) was probably not seen in the first few days of STZ treatment because the fasting blood glucose level of the pre-diabetic group was elevated following the induction of diabetes. The spike in the level of blood glucose seen in this group may be transient and could have decreased if the experimental period were kept for a longer period.

Moreover, the administration of ghrelin for four weeks to normal rats in the pre-diabetic group did not prevent the reduction in body weight after the induction of diabetes in the 5th week. This implies that normal rats pre-treated with ghrelin were not protected from developing DM with body weight loss. Body weight loss is a key feature of STZ-induced DM [70].

In another experiment done to confirm the effect of ghrelin on insulin release, different concentrations of ghrelin were used to stimulate the CRL11065 beta cell line. Ghrelin induced a dose-dependent increase in insulin release from this beta cell line. This observation confirms the report of Adeghate and Ponery in 2002 that ghrelin can stimulate large and significant increases in insulin release from the pancreas [42].

Even though ghrelin treatment did not prevent nor delay the onset of diabetes mellitus in the pre-diabetic, ghrelin-treated group, ghrelin probably increases serum insulin level via the direct stimulation of insulin and enhancement of the proliferation of pancreatic beta cells (Figure 6). A more robust approach that includes modification of parameters such as dose, duration and frequency of the treatment may result in more positive outcomes.

## 5. Conclusions

In conclusion, ghrelin co-localizes with insulin in pancreatic beta cells, where they reside with insulin molecule in secretory granules. The administration of ghrelin increases the number of insulin-secreting cells, which explains the elevated serum insulin level observed at the end of the experiment. Moreover, ghrelin stimulates insulin release from CRL11065 beta cell line. This suggests that ghrelin may have a role in the management of DM.

## Figures and Tables

**Table 1:** List of the primary and secondary antibodies of names and their dilutions.

Primary	Dilution	Secondary	Dilution
Ghrelin (Phoenix Pharmaceuticals, Burlingame, CA, USA)	1:100	RRX FITC (Jackson Laboratories, Bar Harbor, ME, USA)	1:100
Insulin (Dako, Glostrup, Denmark)	1:1000	FITC (Jackson Laboratories, Bar Harbor, ME, USA)	1:100

## Abbreviations

STZ	Streptozotocin
DM	Diabetes mellitus
NS	Normal saline
NGS	Normal goat serum
FBG	Fasting blood glucose
GTT	Glucose tolerance test
EDTA	Ethylenediamine tetra-acetic acid
IHC	Immunohistochemistry
IF	Immunofluorescence
PBS	Phosphate buffered solution
ABC	Avidin-biotin complex
FITC	Fluorescein Isothiocyanate
RRX	Rhodamine Red-X
SD	Standard deviation
T1DM	Type 1 Diabetes Mellitus
T2DM	Type 2 Diabetes Mellitus
β cells	Beta cells/Insulin secreting cells
